# The imbalance between Type 17 T-cells and regulatory immune cell subsets in psoriasis vulgaris

**DOI:** 10.3389/fimmu.2022.1005115

**Published:** 2022-08-30

**Authors:** Jaehwan Kim, Ariana Moreno, James G. Krueger

**Affiliations:** ^1^ Laboratory for Investigative Dermatology, The Rockefeller University, New York, NY, United States; ^2^ Dermatology Section, Veterans Affairs Northern California Health Care System, Mather, CA, United States; ^3^ Department of Dermatology, University of California Davis, Sacramento, CA, United States

**Keywords:** psoriasis, type 17 T-cells, regulator T-cell, type 1 regulatory T-cell, regulatory dendritic cell, immune tolerance, immune homeostasis

## Abstract

Psoriasis vulgaris is a common inflammatory disease affecting 7.5 million adults just in the US. Previously, psoriasis immunopathogenesis has been viewed as the imbalance between CD4^+^ T-helper 17 (Th17) cells and regulatory T-cells (Tregs). However, current paradigms are rapidly evolving as new technologies to study immune cell subsets in the skin have been advanced. For example, recently minted single-cell RNA sequencing technology has provided the opportunity to compare highly differing transcriptomes of Type 17 T-cell (T17 cell) subsets depending on IL-17A *vs*. IL-17F expression. The expression of regulatory cytokines in T17 cell subsets provided evidence of T-cell plasticity between T17 cells and regulatory T-cells (Tregs) in humans. In addition to Tregs, other types of regulatory cells in the skin have been elucidated, including type 1 regulatory T-cells (Tr1 cells) and regulatory dendritic cells. More recently, investigators are attempting to apply single-cell technologies to clinical trials of biologics to test if monoclonal blockade of pathogenic T-cells will induce expansion of regulatory immune cell subsets involved in skin homeostasis.

## Introduction

**Figure 1:**
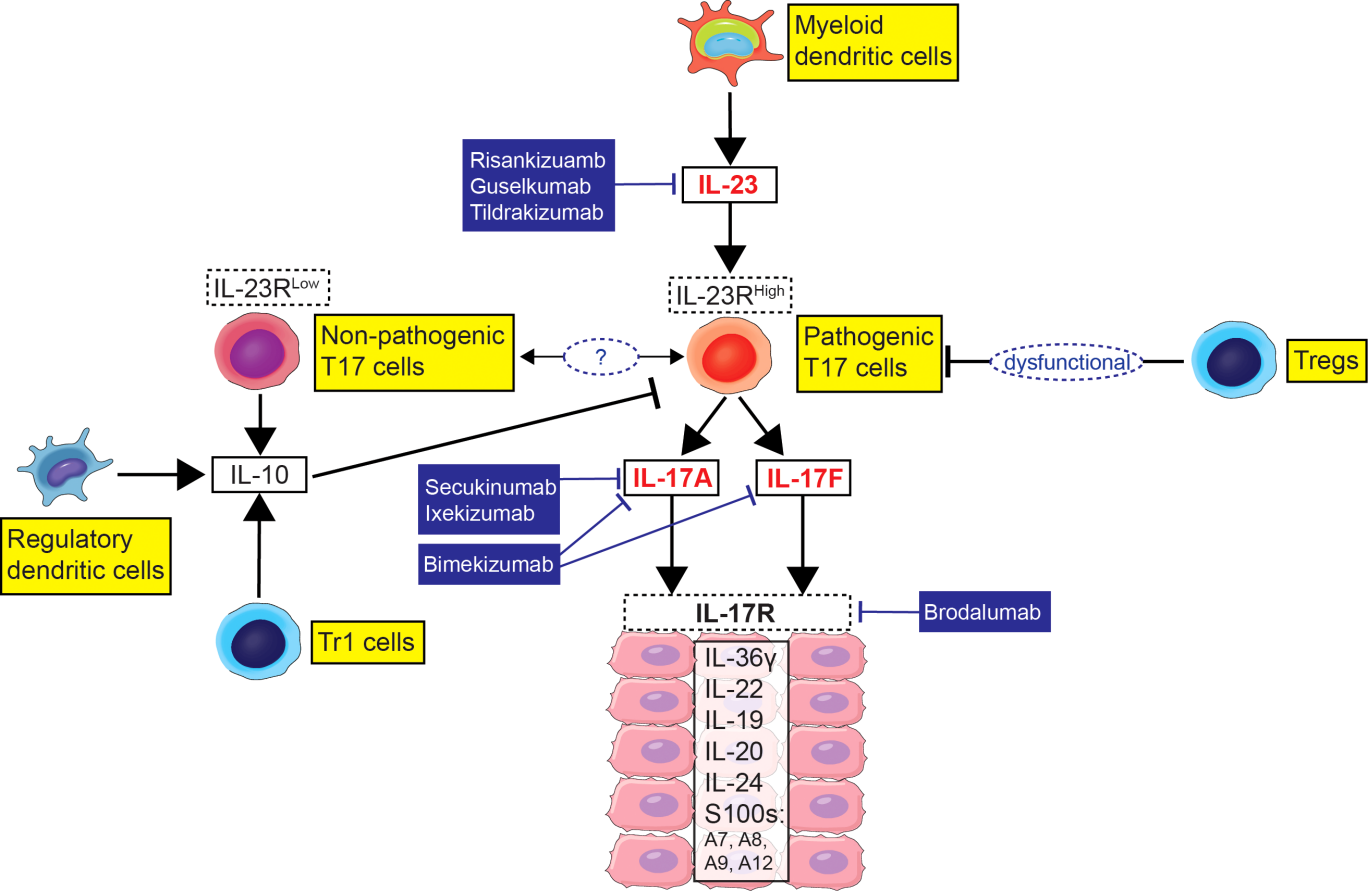
Immune model of the imbalance between Type 17 T-cells (T17 cells) and regulatory immune cell subsets in psoriasis with biologic agents targeting the IL-23/T17 cell axis.

Psoriasis vulgaris is a debilitating chronic inflammatory disease affecting 7.5 million adults just in the US (1). The pathogenic contribution of IL-17 producing T-cells (Type 17 T-cells; T17 cells) in psoriasis is substantiated by the high efficacy of psoriasis biologic treatments targeting IL-17 or IL-23 which regulates T17 cells (**Figure 1**). However, the estimated annual direct health care cost of psoriasis treatment is $63 billion, and it is rapidly increasing with the cost driven by premium-priced biologics (2, 3). Although highly effective, disease clearance of psoriasis biologics targeting the IL-23/T17 cell axis ranges from 12% to 51% (4) and the biologics should be continuously injected at 2-to-12-week intervals to suppress recurrence (5). Psoriasis frequently recurs 12 to 34 weeks after treatment withdrawal, and lifelong therapy is often required to sustain disease remission (6, 7). When moderate-to-severe psoriasis patients were treated with IL-17A monoclonal antibody (secukinumab) for 52 weeks and then stopped the medication, 84% of patients experienced relapse of psoriasis within the next 52 weeks (8). To cure psoriasis without recurrence, we need to further understand how immune tolerance becomes dysfunctional in psoriasis patients and how to restore skin homeostasis after psoriasis treatment.

Previously, dysfunctional immune tolerance in psoriasis has been viewed as the imbalance between T-helper 17 (Th17) cells and regulatory T-cells (Tregs). Th17 cells have been considered the primary pathogenic cells in psoriasis lesions and Tregs have been considered the primary regulatory cells in the skin. It has been reported that while psoriasis patients have normal numbers of circulating Tregs, psoriatic Treg cells are less effective at suppressing alloreactive T-cells compared to Treg cells from normal individuals (9–11). In addition, a lower expression of immune checkpoint molecules in psoriasis lesions than a resolving cutaneous delayed-type hypersensitivity reaction have been reported (12).

**Figure 2:**
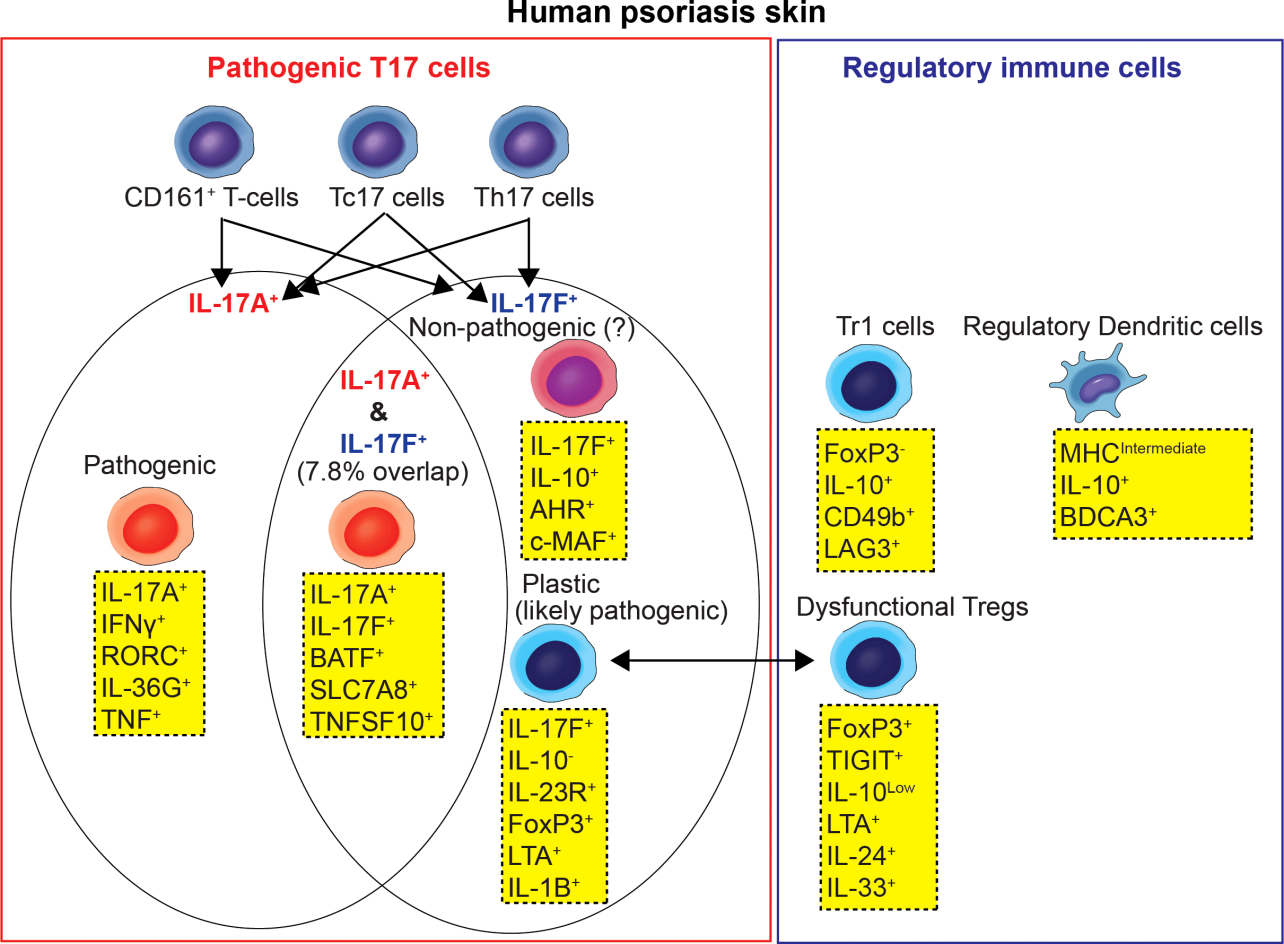
Pathogenic Type 17 T-cell and regulatory immune cell subset network model of human psoriasis skin.

However, current paradigms of dysfunctional immune tolerance in psoriasis are rapidly evolving as new technologies to study immune cell subsets in the skin have been advanced. For example, recently minted single-cell RNA sequencing technology has provided the opportunity to compare the transcriptomes of T-cell subsets that commonly produce IL-17 isoforms (T17 cells) or Tregs. Here, we review a broad spectrum of pathogenic and regulatory immune cell subsets in psoriasis human skin beyond Th17 cells and Tregs (**Figure 2** , **Table 1**). We also reviewed recent clinical trials testing if monoclonal blockade of pathogenic T-cells could induce expansion of regulatory immune cell subsets or cytokines involved in skin homeostasis.

**Table 1:** Key publications reviewed.

Psoriasis T-cell and immune cell subset studies		References
Single-cell transcriptome analyses of human psoriasis skin		(13)
Type 17 T-cells	Tc17 cells	(14–17)
	CD161^+^ T-cells	(18–22)
	Pathogenic versus non-pathogenic T17 cells	(23–33)
Regulatory immune cell subset	Tregs	(9)
	Tr1 cells	(32, 34–37)
	Regulatory DCs	(38, 39)
Clinical trials with skin transcriptome analyses		(40–44)

## T17 cell subsets beyond Th17 cells in psoriasis

### T17 cell subsets

Unlike other inflammatory diseases where CD4^+^ Th17 cells are the main IL-17 producing T-cell subset, CD8^+^ T-cells are also an important source of IL-17 in psoriasis skin (Tc17 cells) (14). Tc17 cells are cytotoxic effector cells that secrete Th17-related cytokines, and they are enriched in psoriasis skin lesions and psoriatic arthritis joints (15, 16). In psoriasis skin, Tc17 cells have been investigated in the context of tissue-resident memory T-cells (T_RM_ cells). Human skin contains many tissue-resident memory T-cells (T_RM_ cells) from childhood, but these cells normally do not produce inflammation due to immune tolerance. However, in the epidermis of psoriasis skin, Tc17 cells expressing the T_RM_ marker CD103 maintain their inflammatory capacity even in resolved psoriasis lesions after effective treatment (17). This may partially explain why psoriasis tends to recur in previously affected areas, rather than new lesions occurring in previously unaffected areas. In psoriatic arthritis joints, the frequency of Tc17 cells were correlated with disease activity and joint damage progression (16).

CD161 is another marker identifying T17 cell subsets in psoriasis skin. CD161^+^ T-cells are present in greater numbers in psoriasis lesional skin than in normal control skin or psoriasis nonlesional skin (18, 19). The greater frequency of CD161^+^ T-cells in the prepsoriatic skin compared to normal skin has been reported, suggesting the role of CD161^+^ T-cells in the initial development of psoriatic lesions (18, 20). Maggi et al. (21) reported that CD161 is a marker of all human T-cell subsets with the ability to produce IL-17, and IL-17-producing cells exclusively originate from naïve CD161^+^ T-cell precursors. Cosmi et al. (22) reported that the majority of CD161^+^ T-cells also expressed CCR6, and the great majority of IL-17-producing cells appeared to be included in the CCR6^+^ fraction (22). However, human psoriasis skin single-cell RNA sequencing (scRNA-seq) data implicated that CD161 may not be an exclusive marker for T17 cells. When the percentages of T17 cells in T-cell subsets were directly compared using human psoriasis skin single-cell RNA sequencing (scRNA-seq) data, T17 cells constituted 2.7% of the CD4^+^ T-cell cluster, 1.3% of the CD8^+^ T-cell cluster, 2.4% of the CD161^+^ T-cell cluster and 0.5% of the Treg cluster (13). In addition, the IL-17 expressing CD161^+^ T-cell cluster included CD8^+^ T-cells in the scRNA-seq data (13), while previous IL-17 producing CD161^+^ T-cells were exclusively studied in CD4^+^ T-cells (21, 22).

## IL-17A^+^ *vs*. IL-17F^+^ T17 cell subsets and pathogenic *vs*. non-pathogenic T17 cell subsets in psoriasis

T17 cells are T-cells that produce the IL-17 isoforms (IL-17A and IL-17F). Previously, the general consensus was that IL-17A and IL-17F were produced by common T17 cells. However, recent scRNA-seq data showed the possibility that IL-17A producing T17 cells and IL-17F producing T17 cells are different T17 cell subsets in human psoriasis skin. Kim et al. (13) conducted scRNA-seq analyses of skin biopsy tissues from 13 psoriasis patients and 5 healthy volunteers and reported that the majority of T17 cells expressed either IL-17A or IL-17F and only 7.8% of T17 cells co-expressed IL-17A/IL-17F in human psoriasis skin. Cutaneous T17 cells displayed highly differing transcriptomes depending on IL-17A *vs*. IL-17F expression and IFNγ *vs*. IL-10 expression (13).

Data derived from murine models of pathogenic IL-17-producing T-cells suggested that T17 cells may exist in two alternative states - pathogenic T17 cells that produce high levels of IL-17 after being stimulated by IL-23 and non-pathogenic (quasi-regulatory) T17 cells that produce high levels of IL-10 (23–30, 45). Recent scRNA-seq data (13) showed that the T17 cell subset that most conforms to current concepts of pathogenic Type 17 T-cells in human psoriasis skin (23, 25, 27, 31) was the IL-17A^+^ IFNγ^+^ T17 cell subset, synthesizing high levels of TNF, IL-26 and IL-36G. These were found mostly within CD8^+^ T-cells that co-express cytotoxic markers (Tc17 cells).

The IL-17F^+^ IL-10^+^ T17 cell subset best fits the description of non-pathogenic T17 cells, however this population constituted only 4% of the overall T17 cells (13). IL-17F^+^ IL10^+^ T17 cells expressed low levels of FoxP3 and high levels of Aryl hydrocarbon Receptor (AHR) and c-Maf. Since AHR interacts with c-Maf to promote the differentiation of Tr1 cells (46), it was suggested that this T17 cell subset may have plasticity to differentiate into Tr1 cells rather than Tregs (13).

The IL-17F^+^ IL-10^-^ T17 cell subset constituted 53% of T17 T-cells and was about 5-fold more frequent than the IL-17A^+^ IFNγ^+^ T17 cell subset (13). This subset has the highest expression of the IL-23 receptor, suggesting it might be the most responsive to IL-23 stimulation and conversion to a pathogenic population. This subset might also be the most responsive to therapeutic IL-23 antagonists because of the higher receptor expression. This subset displayed a different array of pro-inflammatory gene transcripts compared to the IL-17A^+^ IFNg^+^ T17 cell subset (13). For example, the IL-17F^+^ IL-10^-^ T17 cell subset showed the highest expression of GM-CSF, LTA, IL-22, IL-24, and IL-1B among all of the identified T17 cell subsets. In particular, the NFkB-activating cytokines (LTA, IL-1B) are expected to synergize with IL-17A and IL-17F for the induction of many typical “IL-17 pathway” products in keratinocytes that initiate “feed forward” inflammation and help to maintain pathogenic gene expression profiles in psoriasis. In murine models, pathogenic Th17 cells have been seen to trans-differentiate into other defined subsets such as FoxP3^+^ Tregs (32). Since FoxP3 had the highest expression in the IL-17F^+^ IL-10^-^ T17 cell subset among all of the T17 cell subsets, it was suggested that this subset may stem from inflammatory conversion of Tregs (13). We believe that the IL-17F^+^ IL-10^-^ T17 cell subset is also pathogenic, at least in some patients, as dual inhibition of IL-17A and IL-17F produces higher PASI (Psoriasis Area and Severity Index) 100 responses (complete skin clearance) compared to results obtained with antibodies that neutralize IL-17A only (33).

## Dysfunctional Tregs and other regulatory immune cell subsets in psoriasis

### Tregs

Sugiyama et al. (9) reported the dysfunction of psoriatic Tregs by showing the decreased inhibitory capacity of psoriatic Tregs in proliferation assays. Supporting the dysfunction of psoriatic Tregs with single-cell transcriptomics, Kim et al. (13) showed that Treg cluster cells, characterized by their high expression of CD25, FoxP3 and CTLA4, expressed high levels of proinflammatory cytokines such as LTA, IL-24, IL-33 and a T-cell exhaustion marker of TIGIT (47) in psoriasis skin. In addition, IL-17A or IL-17F producing T-cells (T17 cells) were present within the Treg cluster (13). T17 cells within the Treg cluster expressed more IL-17F than IL-17A, and were characterized by high expression of EBI3 and IL-24 (13). In contrast, the expression of IL-10 was low in the Treg cluster.

When the FoxP3^High^ and FoxP3^Low^ subsets within the Treg cluster were compared, the FoxP3^High^ Treg subset expressed high levels of EBI3, a subunit of the new IL-12 family cytokine IL-39 (IL-23p19/EBI3) (48). EBI3 together with IL-12 have been reported to negatively regulate T17 cell-mediated immunity (49). On the other hand, the FoxP3^Low^ Treg subset expressed high levels of IL-33 (13). IL-33 has been reported to change Tregs to T17 cells through a dendritic cell-mediated pathway (50).

### Tr1 cells

Immune checkpoints can be delivered by Foxp3^+^ Treg cells as well as Foxp3^-^ cells, including type 1 regulatory T-cells (Tr1 cells) (32, 34, 35). Tr1 cells secrete large amounts of IL-10 with strong IL-10-dependent suppressive activity, while FoxP3 is expressed at low levels (34, 36). It has been demonstrated that surface expressions of CD49b and LAG-3 are sufficient to identify Tr1 cells (34). The proportion of Tr1 (CD49b^+^ LAG-3^+^) cells in CD3^+^ CD4^+^ T-cells in the blood of psoriasis patients was decreased in psoriasis patients compared with healthy individuals, and the proportion of Tr1 cells decreased as PASI increased (37). It was suggested that decreased proportion of Tr1 cells in the blood of psoriasis patients may allow for excess expansion of psoriasis disease-related T-cells in either lymph nodes or cutaneous compartments (37). Tr1 cells were identified in psoriasis nonlesional skin but not in lesional skin, despite the large increase of T-cells in active lesions (37).

### Regulatory dendritic cells

Human skin contains three main populations of dendritic antigen-presenting cells: Langerhans cells, BDCA-1^+^ DCs, and BDCA-3^+^ DCs, with prior work suggesting the BDCA-3^+^ cells have important regulatory functions (51). Recent scRNA-seq analyses of human psoriasis skin identified a distinct cluster of DCs as ‘semimature DC’. This cluster was characterized by intermediate expression of MHC class II molecules and high expression of IL-10 (13). Among all IL-10 expressing cells in psoriasis skin, 53.5% of IL-10 expressing cells were semimature DCs, and 46.5% of IL-10 expressing semimature DCs in psoriasis skin co-expressed BDCA-3. Overall, the single-cell transcriptome of semimature DCs was consistent with the gene expression profiles of previously described human skin resident BDCA3^+^ regulatory DCs (38). BDCA3^+^ regulatory DCs are a subset of myeloid DCs in normal skin characterized by their BDCA-3 expression, IL-10 production, and expression of negative immune regulators. This population is considered regulatory and contributes to immune tolerance under non-inflammatory conditions (38).

IL-12 producing DC is another candidate for the regulatory DC subset in the context of IL-17-driven inflammation. IL-12 is a heterodimeric protein consisting of p35 (IL-12A) and p40 (IL-12B), and it is mainly expressed by activated dendritic cells and macrophages. Unlike the structurally related pathogenic cytokine IL-23 p19 (IL-23A)/p40 (IL-12B), IL-12 was shown to have a regulatory function by restraining the invasion of IL-17-committed γδ T (γδ T17) cells in an imiquimod-induced psoriasis mouse model (39). However, a different study showed that human CD1c^+^ DCs but not BDCA-3^+^ DCs or other antigen-presenting cells secrete high levels of IL-12 and potently prime cytotoxic T-cell responses (52). In lesional skin of patients with psoriasis, p35 (IL-12A) transcripts were not increased, whereas p40 (IL-12B) and p19 (IL-23A) transcripts were increased (53).

## Therapeutic implications in psoriasis

Today, psoriasis is the most effectively treated T-cell mediated inflammatory disease, as the IL-23/T17 cell axis is the main pathogenic pathway in this disease and numerous biologic agents target this axis (**Figure 1**). Treatments include monoclonal antibodies that neutralize IL-17A/A and IL-17A/F isoforms (secukinumab, ixekizumab), monoclonal antibodies that extend IL-17 blockade to include IL-17F/F (brodalumab, bimekizumab), and monocloncal antibodies that neutralize IL-23, either by binding to the p40 subunit (ustekinumab), or by binding to the p19 subunit (tildrakizumab, guselkumab, risankizumab) (5). Typically, moderate-to-severe disease is treated with monoclonal antibody therapy and 80-90% can be expected to have high-grade improvements in disease (5). Molecular profiling of psoriasis tissue, including the scRNA-seq findings discussed above, has led to new research that seeks the emerging information on the potential plasticity of T17 cells for pathogenic *vs*. regulatory programs:

### IL-17A blockade

When gene-expression profiles of human psoriasis skin were compared between mild versus severe psoriasis, the spectrum of mild to severe psoriasis was defined by a common activation of IL-17 pathway genes, but with key differences in regulatory immune genes (40, 41). Since the expression of immune regulatory genes is high in mild psoriasis, it was hypothesized that treatment response and disease remission in mild psoriasis after IL-17A inhibition might differ from those with severe psoriasis. To test the hypothesis, Kim et al. (42) conducted an exploratory phase II randomized clinical trial (RCT) to test secukinumab (IL-17A inhibitor) for mild-to-moderate psoriasis (NCT03131570). The study showed that the impact of systemic IL-17A inhibition on psoriasis patients’ skin immunity was not confined to only blocking the major cytokine of pathogenic T-cells (IL-17A). This study found that it extended to blocking the entire feed-forward amplification loop of psoriasis inflammation between dendritic cells, T-cells, and keratinocytes. Of note, the gene expression of a keratinocyte stem cell marker [*KRT15* (54)] and cytokines that may promote skin homeostasis [IL-34 (55, 56) and IL-37 (57)] were increased in psoriasis skin after systemic IL-17A inhibition (42).

### IL-23 blockade

T17 cells are regulated by inflammatory CD11c^+^ dendritic antigen-presenting cells (APCs) that over-produce IL-23. The IL-23 expression in inflammatory CD11c^+^ dendritic APCs in psoriasis lesional skin is ~20-fold higher than in APCs in non-lesional psoriatic skin (53). The first observation suggesting that IL-23 blockade may contribute to the restoration of skin homeostasis came from a phase I clinical trial with risankizumab (anti-IL-23p19 monoclonal antibody) (43). In the clinical trial, the administration of single dose of an anti-IL-23p19 antibody resulted in disease clearance for up to 66 weeks in 46% of moderate-to-severe psoriasis patients. A subset of patients attained full disease resolution for up to 16.5 months. In the posttreatment biopsy specimens of those patients, FoxP3 mRNA levels remained high in the resolved psoriasis skin. In a separate study (44), BLIMP-1 (B lymphocyte-induced maturation protein-1) and IL-10 expression within T-cells were found to be increased in resolved psoriasis skin after IL-23 blockade. Since the expression of FoxP3 and IL-10 are high in Tregs, and the expression of BLIMP-1 is essential for the production of IL-10 (58), it was suggested that IL-23p19 inhibition increased either the amount of Treg or their function in resolved psoriatic skin.

These high-grade responses for unexpectedly long periods were achieved by just a single injection of an anti-IL-23p19 monoclonal antibody. Furthermore, structured drug wash-out studies during phase II and III clinical trials of anti-IL-23p19 monoclonal antibodies identified that the majority of patients maintained PASI75 and PASI90 responses for long periods of time after receiving a final dose and stopping further injections (59, 60). In an effort to further understand the immune tolerance mechanism behind the short-term IL-23 blockade associated with long-term disease remission, moderate-to-severe psoriasis clinical trials with scRNA-seq analyses of human skin are currently taking place in the US (NCT04630652).

## Conclusions

Conventional methods of studying the imbalance between T17 cells and regulatory immune cell subsets in human psoriasis skin, requires you to decide which immune cell subsets to target with predetermined markers and then investigate their gene expression profiles and functions in different conditions. However, recent scRNA-seq technology provided the opportunity to compare the gene expression profiles of heterogenous immune cells in the skin without the need to predetermine markers for various cell subsets. For example, in the scRNA-seq dataset, T17 cells can be first defined as IL-17A or IL-17F expressing cells in T-cell clusters, Tregs can be first defined as CD25^+^ FoxP3^+^ CD4^+^ cells, and regulatory immune cells can be first defined as IL-10^+^ expressing immune cell subset clusters. Then, various markers of T17 cells, Treg or regulatory immune cell subsets can be explored in subclusters comparing their differential expressions in different conditions. This new approach enabled us to broaden the category of T17 cells and regulatory immune cell subsets in human psoriasis skin and made it more feasible to understand T-cell plasticity between different T-cell subsets (**Figure 2**, **Table 1**). Thus, we suggest that the complex interplay between T17 cells and regulatory immune cells in the psoriasis immune tolerance network is not confined to just Th17 cells and Tregs. It should be approached with a broad spectrum of immune cell subsets, including Tc17 cells, CD161^+^ T-cells, IL-17A^+^ IFNγ^+^ T17 cell subset, IL17F^+^ IL-10^-^ T17 cell subset, IL17F^+^ IL-10^+^ T17 cell subset, Tregs, Tr1 cells, semimature/BDCA-3^+^ regulatory DCs and cytokines involved in skin homeostasis such as IL-34 and IL-37.

Although the new approach is enabled by the recent scRNA-seq technology, the limitations of the new technology should be carefully considered for translating the results into biologic understandings (61). Current single-cell technologies offer limited number of cells that can be processed at a time and a limited number of mRNA molecules can be captured per cell (62, 63). High variability of gene expression between similar cell types is easily misinterpreted into novel cell subsets (64). Particularly for human skin, technical artifacts of sample processing and computational correction of the artifacts may hinder the reproducibility of key gene detections (65).

Recent clinical trials with gene expression profiling of psoriasis skin revealed promising data supporting the hypothesis that a monoclonal blockade of pathogenic T-cells, such as an IL-17A blockade or an IL-23p19 blockade, may induce expansion of regulatory immune cells subsets or expression of cytokines involved in skin homeostasis (42–44). To test the hypothesis, there are ongoing psoriasis clinical trials of short-term monoclonal antibody treatments to induce long-term disease remission that incorporate scRNA-seq analyses of human skin before/after treatments (NCT04630652). We are hoping to better understand the imbalance between T17 cells and regulatory immune cell subsets through these studies and develop personalized medicine approaches to cure psoriasis without recurrence.

## Author contributions

JK, AM, and JGK have contributed to the drafting or editing of the manuscript and gave their approval for the version submitted. All authors contributed to the article and approved the submitted version.

## Funding

JK and JGK were supported by grant # UL1TR001866 from the National Center for Advancing Translational Sciences (NCATS), National Institutes of Health (NIH) Clinical and Translational Science Award (CTSA) program. JK was supported by the Group for Research and Assessment of Psoriasis and Psoriatic Arthritis (GRAPPA) pilot research grant.

## Conflict of interest

The authors declare that the research was conducted in the absence of any commercial or financial relationships that could be construed as a potential conflict of interest.

## Publisher’s note

All claims expressed in this article are solely those of the authors and do not necessarily represent those of their affiliated organizations, or those of the publisher, the editors and the reviewers. Any product that may be evaluated in this article, or claim that may be made by its manufacturer, is not guaranteed or endorsed by the publisher.

## References

[1] Rachakonda TD Schupp CW Armstrong AW. Psoriasis prevalence among adults in the united states. J Am Acad Dermatol (2014) 70(3):512–6. doi: 10.1016/j.jaad.2013.11.013 24388724

[2] Brezinski EA Dhillon JS Armstrong AW. Economic burden of psoriasis in the united states: A systematic review. JAMA Dermatol (2015) 151(6):651–8. doi: 10.1001/jamadermatol.2014.3593 25565304

[3] Rizvi S Chaudhari K Syed BA. The psoriasis drugs market. Nat Rev Drug Discovery (2015) 14(11):745–6. doi: 10.1038/nrd4763 26514859

[4] Beck KM Sanchez IM Yang EJ Liao W. Profile of tildrakizumab-asmn in the treatment of moderate-to-severe plaque psoriasis: evidence to date. Psoriasis: Targets Ther (2018) 8:49. 10.2147/PTT.S146640 PMC6120577 30214892

[5] Kim J Krueger JG. Highly effective new treatments for psoriasis target the IL-23/Type 17 T cell autoimmune axis. Annu Rev Med (2017) 68:255–69. doi: 10.1146/annurev-med-042915-103905 27686018

[6] Masson Regnault M Shourick J Jendoubi F Tauber M Paul C. Time to relapse after discontinuing systemic treatment for psoriasis: A systematic review. Am J Clin Dermatol (2022) 23(4):433–47. doi: 10.1007/s40257-022-00679-y PMC9055370 35489008

[7] Menter A Strober BE Kaplan DH Kivelevitch D Prater EF Stoff B. Joint AAD-NPF guidelines of care for the management and treatment of psoriasis with biologics. J Am Acad Dermatol (2019) 80(4):1029–72. doi: 10.1016/j.jaad.2018.11.057 30772098

[8] Blauvelt A Langley R Szepietowski J Sirgurgeirsson B Tyring S Messina I. Secukinumab withdrawal leads to loss of treatment responses in a majority of subjects with plaque psoriasis with retreatment resulting in rapid regain of responses: a pooled analysis of two phase 3 trials. J Am Acad Dermatol (2016) 74(5):AB273.

[9] Sugiyama H Gyulai R Toichi E Garaczi E Shimada S Stevens SR. Dysfunctional blood and target tissue CD4+CD25high regulatory T cells in psoriasis: Mechanism underlying unrestrained pathogenic effector T cell proliferation. J Immunol (2005) 174(1):164–73. doi: 10.4049/jimmunol.174.1.164 PMC2903964 15611238

[10] de Boer OJ van der Loos CM Teeling P van der Wal AC Teunissen MB. Immunohistochemical analysis of regulatory T cell markers FOXP3 and GITR on CD4+CD25+ T cells in normal skin and inflammatory dermatoses. J Histochem Cytochem (2007) 55(9):891–8. doi: 10.1369/jhc.6A7119.2007 17478450

[11] Zhang L Yang XQ Cheng J Hui RS Gao TW. Increased Th17 cells are accompanied by FoxP3(+) treg cell accumulation and correlated with psoriasis disease severity. Clin Immunol (2010) 135(1):108–17. doi: 10.1016/j.clim.2009.11.008 20006553

[12] Gulati N Suarez-Farinas M Correa da Rosa J Krueger JG. Psoriasis is characterized by deficient negative immune regulation compared to transient delayed-type hypersensitivity reactions. F1000Res (2015) 4:149. doi: 10.12688/f1000research.6581.1 26236467 PMC4505786

[13] Kim J Lee J Kim HJ Kameyama N Nazarian R Der E. Single-cell transcriptomics applied to emigrating cells from psoriasis elucidate pathogenic versus regulatory immune cell subsets. J Allergy Clin Immunol (2021) 148(5):1281–92. doi: 10.1016/j.jaci.2021.04.021 PMC8553817 33932468

[14] Hijnen D Knol EF Gent YY Giovannone B Beijn SJ Kupper TS. CD8(+) T cells in the lesional skin of atopic dermatitis and psoriasis patients are an important source of IFN-γ, IL-13, IL-17, and IL-22. J Invest Dermatol (2013) 133(4):973–9. doi: 10.1038/jid.2012.456 PMC3835628 23223131

[15] Ortega C Fernández AS Carrillo JM Romero P Molina IJ Moreno JC. IL-17-producing CD8+ T lymphocytes from psoriasis skin plaques are cytotoxic effector cells that secrete Th17-related cytokines. J Leukoc Biol (2009) 86(2):435–43. doi: 10.1189/jlb.0109046 19487306

[16] Menon B Gullick NJ Walter GJ Rajasekhar M Garrood T Evans HG. Interleukin-17+CD8+ T cells are enriched in the joints of patients with psoriatic arthritis and correlate with disease activity and joint damage progression. Arthritis Rheumatol (2014) 66(5):1272–81. doi: 10.1002/art.38376 PMC4158887 24470327

[17] Cheuk S Wikén M Blomqvist L Nylén S Talme T Ståhle M. Epidermal Th22 and Tc17 cells form a localized disease memory in clinically healed psoriasis. J Immunol (2014) 192(7):3111–20. doi: 10.4049/jimmunol.1302313 PMC3962894 24610014

[18] Peternel S Kastelan M. Immunopathogenesis of psoriasis: focus on natural killer T cells. J Eur Acad Dermatol Venereol (2009) 23(10):1123–7. doi: 10.1111/j.1468-3083.2009.03292.x 19453772

[19] Cameron AL Kirby B Fei W Griffiths CE. Natural killer and natural killer-T cells in psoriasis. Arch Dermatol Res (2002) 294(8):363–9. doi: 10.1007/s00403-002-0349-4 12420105

[20] Curry JL Qin J-Z Robinson J Nickoloff BJ. Reactivity of resident immunocytes in normal and prepsoriatic skin using an *ex vivo* skin-explant model system. Arch Pathol Lab Med (2003) 127(3):289–96. doi: 10.5858/2003-127-0289-roriin 12653571

[21] Maggi L Santarlasci V Capone M Peired A Frosali F Crome SQ. CD161 is a marker of all human IL-17-producing T-cell subsets and is induced by RORC. Eur J Immunol (2010) 40(8):2174–81. doi: 10.1002/eji.200940257 20486123

[22] Cosmi L De Palma R Santarlasci V Maggi L Capone M Frosali F. Human interleukin 17-producing cells originate from a CD161+CD4+ T cell precursor. J Exp Med (2008) 205(8):1903–16. doi: 10.1084/jem.20080397 PMC2525581 18663128

[23] Lee Y Awasthi A Yosef N Quintana FJ Xiao S Peters A. Induction and molecular signature of pathogenic TH17 cells. Nat Immunol (2012) 13(10):991–9. doi: 10.1038/ni.2416 PMC3459594 22961052

[24] Gaublomme JT Yosef N Lee Y Gertner RS Yang LV Wu C. Single-cell genomics unveils critical regulators of Th17 cell pathogenicity. Cell (2015) 163(6):1400–12. doi: 10.1016/j.cell.2015.11.009 PMC4671824 26607794

[25] Hu D Notarbartolo S Croonenborghs T Patel B Cialic R Yang TH. Transcriptional signature of human pro-inflammatory TH17 cells identifies reduced IL10 gene expression in multiple sclerosis. Nat Commun (2017) 8(1):1600. doi: 10.1038/s41467-017-01571-8 29150604 PMC5693957

[26] Aschenbrenner D Foglierini M Jarrossay D Hu D Weiner HL Kuchroo VK. An immunoregulatory and tissue-residency program modulated by c-MAF in human TH17 cells. Nat Immunol (2018) 19(10):1126–36. doi: 10.1038/s41590-018-0200-5 PMC6402560 30201991

[27] Wu X Tian J Wang S. Insight into non-pathogenic Th17 cells in autoimmune diseases. Front Immunol (2018) 9:1112. doi: 10.3389/fimmu.2018.01112 29892286 PMC5985293

[28] Xu J Yang Y Qiu G Lal G Wu Z Levy DE. C-maf regulates IL-10 expression during Th17 polarization. J Immunol (2009) 182(10):6226–36. 10.4049/jimmunol.0900123 PMC2834209 19414776

[29] Wu C Yosef N Thalhamer T Zhu C Xiao S Kishi Y. Induction of pathogenic TH17 cells by inducible salt-sensing kinase SGK1. Nature (2013) 496(7446):513–7. doi: 10.1038/nature11984 PMC3637879 23467085

[30] Yang Y Weiner J Liu Y Smith AJ Huss DJ Winger R. T-Bet is essential for encephalitogenicity of both Th1 and Th17 cells. J Exp Med (2009) 206(7):1549–64. doi: 10.1084/jem.20082584 PMC2715092 19546248

[31] Dambacher J Beigel F Zitzmann K De Toni E Göke B Diepolder HM. The role of the novel Th17 cytokine IL-26 in intestinal inflammation. Gut (2009) 58(9):1207–17. 10.1136/gut.2007.130112 18483078

[32] Gagliani N Amezcua Vesely MC Iseppon A Brockmann L Xu H Palm NW. Th17 cells transdifferentiate into regulatory T cells during resolution of inflammation. Nature (2015) 523(7559):221–5. doi: 10.1038/nature14452 PMC4498984 25924064

[33] Reich K Papp KA Blauvelt A Langley RG Armstrong A Warren RB. Bimekizumab versus ustekinumab for the treatment of moderate to severe plaque psoriasis (BE VIVID): efficacy and safety from a 52-week, multicentre, double-blind, active comparator and placebo controlled phase 3 trial. Lancet (2021) 397(10273):487–98. doi: 10.1016/S0140-6736(21)00125-2 33549193

[34] Gagliani N Magnani CF Huber S Gianolini ME Pala M Licona-Limon P. Coexpression of CD49b and LAG-3 identifies human and mouse T regulatory type 1 cells. Nat Med (2013) 19(6):739–46. 10.1038/nm.3179 23624599

[35] Yao Y Vent-Schmidt J McGeough MD Wong M Hoffman HM Steiner TS. Tr1 cells, but not Foxp3+ regulatory T cells, suppress NLRP3 inflammasome activation *via* an IL-10–dependent mechanism. J Immunol (2015) 195(2):488–97. 10.4049/jimmunol.1403225 26056255

[36] Zeng H Zhang R Jin B Chen L. Type 1 regulatory T cells: a new mechanism of peripheral immune tolerance. Cell Mol Immunol (2015) 12(5):566–71. 10.1038/cmi.2015.44 PMC4579656 26051475

[37] Kim J Lee J Gonzalez J Fuentes-Duculan J Garcet S Krueger JG. Proportion of CD4(+)CD49b(+)LAG-3(+) type 1 regulatory T cells in the blood of psoriasis patients inversely correlates with psoriasis area and severity index. J Invest Dermatol (2018) 138(12):2669–72. doi: 10.1016/j.jid.2018.05.021 29890167

[38] Chu CC Ali N Karagiannis P Di Meglio P Skowera A Napolitano L. Resident CD141 (BDCA3)+ dendritic cells in human skin produce IL-10 and induce regulatory T cells that suppress skin inflammation. J Exp Med (2012) 209(5):935–45. doi: 10.1084/jem.20112583 PMC3348099 22547651

[39] Kulig P Musiol S Freiberger SN Schreiner B Gyülveszi G Russo G. IL-12 protects from psoriasiform skin inflammation. Nat Commun (2016) 7:13466. doi: 10.1038/ncomms13466 27892456 PMC5133729

[40] Kim J Oh CH Jeon J Baek Y Ahn J Kim DJ. Molecular phenotyping small (Asian) versus Large (Western) plaque psoriasis shows common activation of IL-17 pathway genes but different regulatory gene sets. J Invest Dermatol (2016) 136(1):161–72. doi: 10.1038/JID.2015.378 PMC4731034 26763436

[41] Kim J Bissonnette R Lee J da Rosa JC Suárez-Farinas M Lowes MA. The spectrum of mild to severe psoriasis vulgaris is defined by a common activation of IL-17 pathway genes, but with key differences in immune regulatory genes. J Invest Dermatol (2016) 136(11):2173–82. 10.1016/j.jid.2016.04.032 27185339

[42] Kim J Lee J Hawkes JE Li X Kunjravia N Rambhia D. Secukinumab improves mild-to-moderate psoriasis: a randomized, placebo-controlled exploratory clinical trial. J Am Acad Dermatol (2022). doi: 10.1016/j.jaad.2022.04.060 PMC11314355 35551962

[43] Krueger JG Ferris LK Menter A Wagner F White A Visvanathan S. Anti-IL-23A mAb BI 655066 for treatment of moderate-to-severe psoriasis: Safety, efficacy, pharmacokinetics, and biomarker results of a single-rising-dose, randomized, double-blind, placebo-controlled trial. J Allergy Clin Immunol (2015) 136(1):116–24.e7. doi: 10.1016/j.jaci.2015.01.018 25769911

[44] Visvanathan S Baum P Vinisko R Schmid R Flack M Lalovic B. Psoriatic skin molecular and histopathologic profiles after treatment with risankizumab versus ustekinumab. J Allergy Clin Immunol (2019) 143(6):2158–69. doi: 10.1016/j.jaci.2018.11.042 30578873

[45] Chalmin F Mignot G Bruchard M Chevriaux A Végran F Hichami A. Stat3 and gfi-1 transcription factors control Th17 cell immunosuppressive activity *via* the regulation of ectonucleotidase expression. Immunity (2012) 36(3):362–73. 10.1016/j.immuni.2011.12.019 22406269

[46] Apetoh L Quintana FJ Pot C Joller N Xiao S Kumar D. The aryl hydrocarbon receptor (AhR) interacts with c-maf to promote the differentiation of IL-27-induced regulatory type 1 (TR1) cells. Nat Immunol (2010) 11(9):854. 20676095 10.1038/ni.1912 PMC2940320

[47] Ostroumov D Duong S Wingerath J Woller N Manns MP Timrott K. Transcriptome profiling identifies TIGIT as a marker of T-cell exhaustion in liver cancer. Hepatology (2021) 73(4):1399–418. doi: 10.1002/hep.31466 32716559

[48] Wang X Wei Y Xiao H Liu X Zhang Y Han G. A novel IL-23p19/Ebi3 (IL-39) cytokine mediates inflammation in lupus-like mice. Eur J Immunol (2016) 46(6):1343–50. doi: 10.1002/eji.201546095 PMC11334612 27019190

[49] Chung Y Yamazaki T Kim B-S Zhang Y Reynolds JM Martinez GJ. Epstein Barr Virus-induced 3 (EBI3) together with IL-12 negatively regulates T helper 17-mediated immunity to listeria monocytogenes infection. PloS Pathog (2013) 9(9):e1003628. doi: 10.1371/journal.ppat.1003628 24068935 PMC3777861

[50] Park SH Jung HJ Kim TS. IL-33 changes CD25(hi) tregs to Th17 cells through a dendritic cell-mediated pathway. Immunol Lett (2020) 218:5–10. doi: 10.1016/j.imlet.2019.12.003 31863784

[51] Guttman-Yassky E Nograles KE Krueger JG. Contrasting pathogenesis of atopic dermatitis and psoriasis–part II: immune cell subsets and therapeutic concepts. J Allergy Clin Immunol (2011) 127(6):1420–32. doi: 10.1016/j.jaci.2011.01.054 21419481

[52] Nizzoli G Krietsch J Weick A Steinfelder S Facciotti F Gruarin P. Human CD1c+ dendritic cells secrete high levels of IL-12 and potently prime cytotoxic T-cell responses. Blood (2013) 122(6):932–42. doi: 10.1182/blood-2013-04-495424 23794066

[53] Lee E Trepicchio WL Oestreicher JL Pittman D Wang F Chamian F. Increased expression of interleukin 23 p19 and p40 in lesional skin of patients with psoriasis vulgaris. J Exp Med (2004) 199(1):125–30. doi: 10.1084/jem.20030451 PMC1887731 14707118

[54] Garza LA Yang C-C Zhao T Blatt HB Lee M He H. Bald scalp in men with androgenetic alopecia retains hair follicle stem cells but lacks CD200-rich and CD34-positive hair follicle progenitor cells. J Clin Invest (2011) 121(2):613–22. doi: 10.1172/jci44478 PMC3026732 21206086

[55] Lelios I Cansever D Utz SG Mildenberger W Stifter SA Greter M. Emerging roles of IL-34 in health and disease. J Exp Medicine (2020) 217(3):e20190290. doi: 10.1084/jem.20190290 PMC7062519 31940023

[56] Farrag DA Asaad MK Ghobrial CK. Evaluation of IL-34 in psoriasis and psoriatic arthritis patients: correlation with disease activity and severity. Egyptian Rheumatologist (2017) 39(1):25–31.

[57] Rønholt K Nielsen AL-L Johansen C Vestergaard C Fauerbye A López-Vales R. IL-37 expression is downregulated in lesional psoriasis skin. ImmunoHorizons (2020) 4(11):754–61. doi: 10.4049/immunohorizons.2000083 33239358

[58] Neumann C Heinrich F Neumann K Junghans V Mashreghi MF Ahlers J. Role of blimp-1 in programing Th effector cells into IL-10 producers. J Exp Med (2014) 211(9):1807–19. doi: 10.1084/jem.20131548 PMC4144744 25073792

[59] Papp KA Blauvelt A Bukhalo M Gooderham M Krueger JG Lacour JP. Risankizumab versus ustekinumab for moderate-to-Severe plaque psoriasis. N Engl J Med (2017) 376(16):1551–60. doi: 10.1056/NEJMoa1607017 28423301

[60] Gordon K Blauvelt A Foley P Song M Wasfi Y Randazzo B. Efficacy of guselkumab in subpopulations of patients with moderate-to-severe plaque psoriasis: a pooled analysis of the phase III VOYAGE 1 and VOYAGE 2 studies. Br J Dermatol (2018) 178(1):132–9. 10.1111/bjd.16008 28940259

[61] Ascensión AM Araúzo-Bravo MJ Izeta A. Challenges and opportunities for the translation of single-cell RNA sequencing technologies to dermatology. Life (2022) 12(1):67. 35054460 10.3390/life12010067 PMC8781146

[62] Stegle O Teichmann SA Marioni JC. Computational and analytical challenges in single-cell transcriptomics. Nat Rev Genet (2015) 16(3):133–45. doi: 10.1038/nrg3833 25628217

[63] Hou W Ji Z Ji H Hicks SC. A systematic evaluation of single-cell RNA-sequencing imputation methods. Genome Biol (2020) 21(1):218. doi: 10.1186/s13059-020-02132-x 32854757 PMC7450705

[64] Andrews TS Hemberg M. False signals induced by single-cell imputation. F1000Res (2018) 7:1740. doi: 10.12688/f1000research.16613.2 30906525 PMC6415334

[65] Ascension AM Arauzo-Bravo MJ Izeta A. The need to reassess single-cell RNA sequencing datasets: the importance of biological sample processing. F1000Res (2021) 10:767. doi: 10.12688/f1000research.54864.2 35399227 PMC8984215

